# Facilitators and Barriers of Incremental Innovation by Fixed Dose Combinations in Cardiovascular Diseases

**DOI:** 10.3390/jcdd11070186

**Published:** 2024-06-21

**Authors:** András Inotai, Zoltán Kaló, Zsuzsanna Petykó, Kristóf Gyöngyösi, Derek T. O’Keeffe, Marcin Czech, Tamás Ágh

**Affiliations:** 1 Center for Health Technology Assessment, Semmelweis University, 1091 Budapest, Hungary; inotai.andras@semmelweis.hu (A.I.); zsuzsanna.petyko@syreon.eu (Z.P.); kristof.gyongyosi@syreon.eu (K.G.); 2 Syreon Research Institute, 1142 Budapest, Hungary; tamas.agh@syreon.eu; 3 School of Medicine, University of Galway, H91TK33 Galway, Ireland; derek.okeeffe@universityofgalway.ie; 4 Department of Pharmacoeconomics, Institute of Mother and Child, 01-211 Warsaw, Poland; marcin.czech@pw.edu.pl; 5 Medication Adherence Research Group, Center for Health Technology Assessment and Pharmacoeconomic Research, University of Pécs, 7623 Pécs, Hungary

**Keywords:** incremental innovation, fixed dose combination, polypill, adherence, pricing, real world data, cardiovascular diseases, medicine, reimbursement

## Abstract

Despite the availability of affordable pharmaceuticals treating cardiovascular diseases (CVDs), many of the risk factors remain poorly controlled. Fixed-dose combinations (FDCs), a form of incremental innovation, have already demonstrated improvements over combinations of single medicines in adherence and hard clinical endpoints. Nevertheless, there are many barriers related to the wider use of FDCs in CVDs. Our aim was to identify these barriers and explore system-level facilitators from a multi-stakeholder perspective. Identified barriers include (i) hurdles in evidence generation for manufacturers, (ii) limited acceptance of adherence as an endpoint by clinical guideline developers and policymakers, (iii) limited options for a price premium for incremental innovation for healthcare payers, (iv) limited availability of real-world evidence, and (v) methodological issues to measure improved adherence. Initiatives to standardize and link healthcare databases in European countries, movements towards improved patient centricity in healthcare, and extended value assessment provide opportunities to capture the benefits of FDCs. Still, there is an emerging need to facilitate the generalizability of sporadic clinical evidence across different FDCs and to improve adherence measures. Finally, healthcare payers need to be convinced to pay a fair premium price for the added value of FDCs to incentivize incremental innovation in CVD treatment.

## 1. Introduction

In recent decades, pharmaceutical research and development (R&D) has been highly successful in the treatment of cardiovascular diseases (CVDs). As a consequence, significant therapeutic experience has been accumulated with cardiovascular (CV) medicines, accompanied by well-known safety profiles and affordable price tags. In comparison with other disease areas (e.g., oncology), the unmet need for general CV patient populations is relatively small [1]. Due to off-patent standard therapies and regulators’ expectations for large-scale clinical trials with over ten thousand patients, the return on investment for de novo revolutionary innovation in CVDs has become limited [2], and so innovators have been focusing only on narrow patient populations in niche areas, such as amyloid cardiomyopathy [3,4].

Despite the wide treatment armamentarium, CVDs still represent the largest global disease burden for adults [5,6,7]. CVD is the leading cause of death globally [8], and due to the rising prevalence of CVDs, the health burden will grow in the future. Many CVD risk factors remain poorly controlled. The silent course of the CVD, complexity of treatment sequencing, increasing rates of comorbidities (e.g., diabetes, obesity, and autoimmune diseases), and suboptimal adherence are important factors limiting the translation of clinical trial efficacy into real-world effectiveness at the population level. In the absence of de novo innovation for the general CV population, there are two potential ways forward: First, the synergistic effect of co-administering medicines with different mechanisms of action has improved therapeutic outcomes, e.g., in hypertension, the combination of diuretics, calcium channel blockers, beta-blockers, and renin–angiotensin–aldosterone system blockers has been proved to be effective in reducing major adverse cardiac events (MACEs) [9,10]. The second way forward is the incremental or evolutionary innovation of existing medicines to create value-added or repurposed medicines. There are three drug repurposing models: (i) drug repositioning refers to finding new indications or applying a medicine in a new patient population; (ii) drug reformulation is a process in which the pharmaceutical formulation of a product is modified to gain new attributes; finally, (iii) the combination of established medicines into a single pill. Fixed dose combinations (FDCs) of monocomponents in the same indication, or polypills of medicines from different indications, have the potential to deliver additional value to patients, healthcare professionals, and healthcare payers [11]. The selection of active product ingredients (APIs) for combination should be based on the following considerations: (1) the APIs in the combination should have different and complementary mechanisms of action, (2) their pharmacokinetics should not be widely different, and (3) they should not have supra-additive toxicity compared to monotherapy [12]. For example, better blood pressure control and a reduction in low-density lipoprotein concentration have been demonstrated with the use of FDCs in secondary prevention of CVDs [13]. Importantly, the WHO has recently incorporated FDCs in its Essential Medicines List [14]. Due to the high global prevalence of CVDs, incremental innovation of marketed medicines has more potential to reduce the global burden of CVDs than de novo innovation in niche CV patient populations.

Despite the availability of numerous effective and well-tolerated pharmacotherapies in CVDs, their potential benefits are often not fully realized due to medication non-adherence [15]. Moreover, the necessity to take multiple medications for CVD combination therapies may negatively impact adherence. Approximately half of the patients on chronic pharmaceutical therapy fail to follow their long-term medication regimens [16], which is amplified by the rising prevalence of multimorbidity within aging populations [17,18,19]. As a potential solution, simplified treatment regimens and reduced pill burden through FDCs can improve medication adherence among CVD patients [20].

The impact of non-adherence is especially critical for those patients for whom limited disease control is associated with negative clinical events and increased mortality [21,22,23,24,25,26]. A recent meta-analysis––which included observational (cohort or case–control) studies with risk estimates for cardiovascular events, strokes, or all-cause mortality related to lipid-lowering, antihypertensive, antidiabetics, and antithrombotic agents––highlighted that a 20% increase in adherence to CV medication is associated with a 9% reduction in the risk of CV events, a 16% reduction in the risk of stroke, and a 10% reduction in the risk of all-cause mortality [27]. Conversely, in Europe, 9% of all CV events can be associated with poor medication adherence [28]. Real-world studies on CVDs have demonstrated that FDCs, by improving adherence, are associated with a significant reduction in all-cause mortality (RR: 0.90; 95% CI: 0.81–1.00) and a non-significant reduction in the risk of major cardiac events (MACEs) (RR: 0.85, 95% CI: 0.70–1.02) [29]. Obviously, these indicators may vary for CV diseases (such as coronary heart diseases, chronic heart failure, or stroke) and subpopulations with different baseline risks.

Medication non-adherence in CVDs also leads to substantial healthcare costs due to increased utilization of preventable healthcare services, such as medication use, emergency department visits, outpatient visits, and hospital admissions [30]. Such cost increases become particularly critical when considering that expenditures related to CVD represent up to 7.6% of total healthcare spending in the European Union, partly driven by medication non-adherence [31]. This suggests that, among other potential adherence interventions, FDCs offer a promising strategy to mitigate the healthcare burden associated with CVDs [20,32]. The annual savings associated with changing combination antihypertensive therapy to FDCs in Poland was estimated to be EUR 12.3 million from the public payer’s perspective and an additional EUR 5.0 million from the patients’ perspective. Apart from the immediate savings in pharmaceutical costs, the broadened use of FDCs was also expected to improve the patients’ adherence and non-drug costs (e.g., avoided complications) [33].

Finally, the applicability of lower doses due to the improved therapeutic effect of FDCs may be translated to potentially lower collateral effects and reduced utilization and cost of the API.

Potential disadvantages of FDCs include limited flexibility in dosing (both during the titration period and during the maintenance treatment period), hindering personalized medicines, and, compared to administering only one monocomponent, potentially increased chances of adverse drug effects and interactions [34].

Several publications describe how medication adherence at the patient level can be facilitated by healthcare professionals (e.g., physicians, pharmacists, and nurses) with the support of clinical societies and patient advocacy groups [35,36]. However, there is limited information about how policymakers and healthcare payers at the health system level can advocate incremental innovation of health technologies—such as FDCs or digital health solutions—with the potential to improve therapeutic adherence.

This perspective paper aims to describe the barriers preventing the increased utilization of FDCs in CVDs and, based on the identified barriers, to provide system-level policy solutions and recommendations from a multi-stakeholder perspective.

## 2. System Level Barriers to Advocate FDCs

In order to explore system-level barriers towards the development and use of FDCs to improve medication adherence, it is important to understand what differentiates incremental innovation of widely used medicines from other types of pharmaceutical innovations from the perspective of regulators and healthcare payers.

As described in Table 1, the patentability of FDCs is fairly limited compared to other types of pharmaceutical R&D. HTA bodies and healthcare payers treat FDCs similarly to generic or biosimilar medicines. They do not expect health gain compared with the combination of monocomponents, which has to be rewarded with a price premium. In theory, healthcare payers are not against paying a fair price premium for a reduction in mortality or an improvement in quality of life if manufacturers are able to prove these benefits at product launch from randomized clinical trials (RCTs). The current approach of macro-level decisions is associated with several barriers to the extended development and use of FDCs in CVDs.

### 2.1. Barrier #1: Hurdles in Evidence Generation for Innovators of FDCs

When comparing FDCs to combination therapies in separate pills, it is difficult to separate the improved therapeutic effect coming (1) from the synergistic effect of co-administering single medicines with different mechanisms of action, or (2) from the benefit of combining established medicines into a single pill. Investment in evidence generation related to the added value of the single pill formulation is a dilemma for the first movers due to the limited patentability, as once the evidence is in the public domain, other manufacturers will have the opportunity to free-ride on the first mover’s investment and develop their own FDCs with the same components.

As a consequence of the limited patentability, pharmaceutical companies have to limit the R&D budget to test the benefits of FDCs of off-patent medicines in prospective randomized controlled trials (RCTs). Even if they are willing to invest a development budget comparable to de novo investigational medicines, it is not possible to detect adherence improvement of FDCs in blinded RCTs. Therefore, clinical differentiation of FDCs in RCTs has to be based on hard clinical endpoints, which necessitates the inclusion of thousands of patients with long-term follow-up. Improved medication adherence (and related improvement in hard clinical endpoints) can potentially be measured in observational studies. Recently, there has been increasing evidence that biochemical urine testing is a promising tool for measuring adherence in multiple chronic diseases, including diabetes and mental disorders [37,38,39]. However, the generation of real-world evidence is possible only after the wide-scale use of FDCs.

In conclusion, the benefits of FDCs can be proven only in highly expensive, large-scale RCTs and real-world evidence is available only after the reimbursement of FDCs by healthcare payers.

### 2.2. Barrier #2: Limited Acceptance of Adherence as an Endpoint by Clinical Guideline Developers and Policymakers

Certain clinical guidelines already recognize the importance of FDCs in reducing pill burden and improving adherence in the pharmacotherapy of CVDs. Specifically, the ESH (European Society of Hypertension) guidelines [40] emphasize treatment initiation with FDCs rather than the administration of separate monocomponents for most patients with hypertension. Similarly, the ACC/AHA (American College of Cardiology/American Heart Association) guidelines [41] recommend the same approach for patients with stage 2 hypertension, those with blood pressure more than 20/10 mm Hg above their target blood pressure, and Black patients.

Nevertheless, the integration of FDCs into CV clinical guidelines is still in its early stages and not yet comprehensive. Despite the significant potential of FDCs in enhancing medication adherence, they face four main challenges in gaining extensive recognition as a valid strategy for clinical improvement within clinical guidelines. First, the development of evidence-based guidelines heavily focuses on hard clinical endpoints, such as mortality and MACEs, thus typically not considering the impact of FDCs on adherence as a valid clinical endpoint. Second, the assertion that FDCs improve medication adherence lacks robust support from RCTs due to the aforementioned reasons, hindering the acceptance of adherence benefits as a legitimate clinical improvement [42]. Third, the lack of regulatory recommendations on standardized definitions, measurement methods, and reporting protocols of medication adherence, both in RCTs and in real-world data analysis, complicates the assessment of adherence interventions, including FDCs [43,44]. Finally, various other factors beyond the formulation of medications can significantly influence medication adherence [16,45,46]. These factors underscore the complexity of enhancing adherence and suggest that a multifaceted approach is necessary to comprehensively address this issue.

### 2.3. Barrier #3: Limited Options for Price Premium for Incremental Innovation by Healthcare Payers

In the financing protocol for treatments of CVDs, the first-line therapies are off-patent medicines. After the patent expiry, the primary expectation of healthcare payers is the generation of savings from generic price erosion, and they devote limited attention to health outcomes. Payers routinely apply internal reference pricing (IRP) for medicines in the retail sector or single-criterion tenders with a ‘lowest-price-wins’ decision rule in the hospital sector [47,48]. Pharmaceutical price regulators, healthcare payers, and procurement bodies set up similarly conservative pricing rules for FDCs, which permit discount prices for FDCs compared with the aggregated price level of monocomponents; in other words, they expect that ‘1 + 1 < 2’. Some payers even maximize the price of FDCs according to the higher-priced monocomponent (’1 + 1 = 1’ or even ‘1 + 1 + 1 = 1’) [33] and hope that manufacturers of FDCs accept such an unfair pricing rule for a potentially higher market share compared with monocomponents.

Exemptions from IRP or a modest price premium are more realistic in areas with high unmet needs, such as oncology or pediatric indications. Based on a series of structured interviews with health policy experts from Ireland, the price potential of FDCs is still not sufficient to incentivize investment in R&D [49]. A good example of that is a polypill called TRINOMIA (containing acetylsalicylic acid, atorvastatin, and ramipril) which is used for the secondary prevention of cardiovascular accidents. Our interviewees highlighted that, despite the substantial added therapeutic value of the polypill, they expected the medicines not to receive a price premium [50]. Based on the reimbursement price of TRINOMIA in Ireland, it was priced slightly higher than the most expensive monocomponent, atorvastatin (EUR 13.11 and EUR 12, respectively), for a 28-day treatment period [51,52]. Additionally, based on the Irish Pharmaceutical Healthcare Association’s 2021–2025 Framework Agreement, a yearly price reduction resulted in an even lower price for TRINOMIA (EUR 12.83) as of the 1st of March 2022 [53]. The negative incentivizing effect of such pricing policies is well demonstrated by the fact that the pivotal trial for TRINOMIA (SECURE trial) was funded by the EU [54,55]. With more adaptive pricing policies and a better price potential, a local pharmaceutical company might have been incentivized to run a trial for the FDC and supply the medicine in Ireland. Our interviews revealed that a similar pricing structure is true for Poland, where the price is usually set to the price level of the highest-priced monocomponent (‘1 + 1 = 1’) [33]. Our experts highlighted that even after the expected future revision of the Polish legislation, the full price may not reach the sum of the two monocomponents’ prices (i.e., ‘1 + 1 < 2’), and they see that this discourages investment in FDCs. Therefore, price regulators, healthcare payers, and procurement bodies do not financially incentivize the added value of improved therapeutic adherence generated by incremental innovation.

Another potential reason why payers are reluctant to pay a price premium for FDCs (with their main benefit being improved adherence) is the multifactorial nature of the issue of non-adherence. As often argued, in addition to therapy-related factors, non-adherence is also influenced by social and economic, disease-related, patient-related, and healthcare-system-related factors. Hence, according to the argument of some payers, isolating and attributing improved adherence solely to the pharmaceutical product itself may be misleading. As an example, one might argue that promotional activities of sales representatives of pharmaceutical companies may have an impact on medication adherence, therefore, payers, similarly to developers of clinical guidelines, also prefer the hard clinical endpoints over medication adherence.

### 2.4. Barrier #4: Limited Availability of Real-World Evidence

Pathways of CVD patients and their long-term outcomes (i.e., mortality and MACEs) can potentially be followed in payer’s databases in countries with a single healthcare payer. However, the accessibility to individualized patient records is often limited to researchers, even in an anonymized format, and missing or poor-quality data also limits the potential of capturing the benefits of FDCs in payer’s databases.

In countries with multiple payers, where healthcare financing is fragmented, the patient pathways may not be reliably traceable.

### 2.5. Barrier #5: Methodological Issues to Measure Improved Adherence

The lack of consensus on how to calculate medication adherence may also complicate the analysis of prescription-refill databases. In the case of polypharmacy, complexity arises from the need to integrate the dosing and timing schedules of each drug as their effects merge into a single treatment regimen [56]. This may pose a challenge for retrospective database analyses attempting to assess real-world adherence improvements with FDCs compared with the combination of monocomponents.

## 3. Recommended Solutions

While the extended use of FDCs at the level of individual CV patients has to be supported by healthcare professionals, it is of utmost importance to change the entire healthcare ecosystem with the involvement of different stakeholders at the macro level.

Dissemination of evidence about the clinical and economic benefits of FDCs has to be facilitated to different stakeholders, including macro-level decision makers, such as regulators and healthcare payers, clinical societies and patient organizations.

Policymakers have to support the extended use of FDCs in CVDs by different measures. FDCs should be included in clinical guidelines and financing protocols at a preferred first-line position. Minimum quotas for the percentage of FDCs in selected CVDs and financial and other incentives to physicians for the prescription of FDCs need to be considered.

Clinical societies and regulators should create consensus about commonly accepted adherence measurement and calculation methods and reporting guidelines [57,58]. If all adherence studies apply the same measures, meta-analyses can be conducted to strengthen the evidence base of adherence-improving technologies. With the increasing evidence about the benefits of FDCs in improving adherence and reducing mortality and MACEs, additional secondary research is needed to explore the generalizability of risk-reduction estimates to future FDCs and also to explore the effect size in different CV diseases and subpopulations with different baseline risks.

Digitalization of medical records and recent initiatives to both standardize and link big data in European countries [59,60], accompanied by current advances in artificial intelligence methodologies, create a unique opportunity to generate real-world evidence (RWE) about the benefits of FDCs in reducing MACEs and mortality, as well as on reduced collateral effects and lower overall API utilization due to potentially lower doses.

The accessibility of payers’ databases to researchers has to be improved, partly because the RWE about the benefits of FDCs would be more acceptable for payers and HTA bodies if the evidence is generated in public ‘non-biased’ databases governed by healthcare payers.

Finally, the added value of FDCs should be acknowledged and incentivized by healthcare payers through new payment models, revising the current conservative pricing models, primarily based on internal reference pricing, into a formula that allows a fair premium price for incremental health gain. However, HTA bodies and healthcare payers cannot expect RCT evidence about the health gain of FDCs before pricing and reimbursement decisions, but they should rely on generalized evidence about the potential reduction in mortality and MACEs by FDCs, and/or they should facilitate ex-post evidence-generation methods based on real-world data, such as coverage with evidence-generation schemes.

## 4. Conclusions

From the societal and industry policy perspectives, the cost of incremental innovation is significantly less than the innovation of de novo medicines. The magnitude of the real-world benefits of FDCs is similar to new medicines, with less uncertainty about safety. FDCs have the potential to improve patient centricity in healthcare, which is an important attribute of extended value assessment. In conclusion, incremental innovation of FDCs in CVDs may be more cost-effective than the development of original medicines [61]. The confirmation of the positive impact of FDCs on adherence has been reinforced recently by the inclusion of these treatments (FDCs and polypills) in the WHO Essential Medicines List [14].

## Figures and Tables

**Table 1:** Current approach of healthcare regulators and policymakers for different types of innovative pharmaceutical technologies.

	Types of Pharmaceutical Innovation				
	Disruptive Pharmaceutical Technologies	Revolutionary Technologies (First-In-Class Medicines)	Incremental Innovation of Medicines with Known Mechanism of Action (Me-Too Medicines)	Incremental Innovation of Widely Used Medicines (e.g., Fixed Dose Combinations)	Non-Innovative Generic or Biosimilar Medicines
Patentability	full	full	full	fairly limited	no
Expectations of health technology assessment (HTA) bodies and payers about health gain	Significant health gain based on superiority clinical trials	Health gain based on superiority clinical trials	Option A: equivalent health gain based on non-inferiority clinical trials Option B: minor health gain based on superiority clinical trials	Option A: equivalent health gain based on bioequivalence of short-term non-inferiority studies Option B: minor health gain based on superiority clinical trials	Equivalent health gain based on bioequivalence study
Acceptability of real-world evidence by bodies and payers	Acceptable ex-ante or ex-post (e.g., coverage with evidence development)	Acceptable ex-ante or ex-post (e.g., coverage with evidence development)	Acceptable ex-ante or ex-post (e.g., coverage with evidence development)	Acceptable ex-ante	No acceptance
Typical pricing approach by healthcare payers	Free pricing; benchmark pricing; value-based pricing (major price premium)	Value-based pricing (minor or major price premium)	Option A: therapeutic reference pricing Option B: value-based pricing (minor price premium)	Option A: internal reference pricing Option B: value-based pricing (minor price premium)	Internal reference pricing
